# Efficacy and safety of Biqi capsule in the treatment of knee osteoarthritis: A protocol of a randomized controlled trial

**DOI:** 10.1097/MD.0000000000025476

**Published:** 2021-04-23

**Authors:** YaZhou Zhou, WenGang Wang, Ke Tian, Hui Huang, Mengrui Jia

**Affiliations:** a Henan Medical College; b The First Affiliated Hospital of Zhengzhou University, Zhengzhou, Henan Province, China.

**Keywords:** Biqi capsule, knee osteoarthritis, protocol, randomized controlled trial

## Abstract

**Background:**

Knee osteoarthritis (KOA) is a chronic and degenerative bone and joint disease, with KOA, cartilage degeneration, destruction and subchondral bone remodeling as the main pathological features. Its clinical symptoms are knee pain, swelling, limited activity, and long course of disease can cause joint deformities. At present, the early treatment of Western medicine is mainly the use of nonsteroidal drugs for anti-inflammation and removing pain, but because the efficacy of these drugs is unstable, the disease is easy to repeat after treatment, and the clinical effect is not good. Although Biqi capsule has advantages in the treatment of KOA, there is a lack of standard clinical studies to verify it, so the purpose of this randomized controlled study is to evaluate the efficacy and safety of Biqi capsule in the treatment of KOA.

**Methods:**

This is a prospective randomized controlled trial to study the efficacy and safety of Biqi capsule in the treatment of KOA. The patients were randomly divided into a treatment group and a control group according to 1:1. Among them, treatment group: Biqi capsule combined with diclofenac sodium sustained release tablets; Control group: Diclofenac sodium sustained-release tablets alone. Both groups were treated with standard treatment for 2 weeks and were followed up for 30 days to pay attention to the efficacy and safety indexes. Observation indicators included: the Western Ontario and McMaster Universities Osteoarthritis Index (WOMAC), Hospital for Special Surgery Knee Score (HSS), liver and kidney function, adverse reactions, and so on. SPSS 25.0 software is used for data analysis.

**Discussion:**

This study will evaluate the efficacy and safety of Biqi capsule in the treatment of KOA, and the results of this experiment will provide a clinical basis for Biqi capsule in the treatment of KOA.

**Trial registration:**

OSF Registration number: DOI 10.17605/OSF.IO/6HB9D

## 1 Introduction

Knee osteoarthritis (KOA) is a common clinical chronic arthritis. Its pathological features are mainly destruction of articular cartilage and hyperplasia of bone, and its clinical manifestations are recurrent joint pain and progressive limitation of joint motion.^[1]^ KOA is closely related to age, obesity, occupation and trauma, and so on,^[2,3]^ and has a high incidence and disability rate,^[4,5]^ it mainly occurs in the middle-aged and elderly people, also known as senile arthritis. Statistics show that,^[6]^ more than 50% of people over the age of 60 have radiographic signs of osteoarthritis, and more than 35% have corresponding symptoms. The incidence of KOA is high, the cost of surgical treatment is high, the long-term effect is not significant, and the recurrence rate is high, which seriously affects the work and life of patients.

Nonsteroidal anti-inflammatory drugs (NSAIDs) are effective in the treatment of inflammatory pain,^[7]^ which is widely used in the treatment of KOA,^[8]^ diclofenac sodium is a common nonsteroidal anti-inflammatory drug preparation in the treatment of KOA. By inhibiting the activity of cyclooxygenase, it reduces the production of prostaglandin, inhibits the activation and differentiation of lymphocytes, and inhibits the production of white blood cells and bradykinin,^[9]^ showing good anti-inflammatory and analgesic effects.^[10]^ However, the efficacy of these drugs is unstable, and the disease is prone to recurrent attacks, which makes it difficult to effectively control the progress of the disease.^[11]^

In recent years, the application of traditional Chinese medicine in KOA has been widely recognized.^[12,13]^ Traditional Chinese medicine believes that the basic pathogenesis of KOA is based on deficient root and excessive superficial, deficiency of liver and kidney qi and blood is the essence, wind, cold and dampness, blood stasis and phlegm, stagnation and accumulation of qi, resulting in obstruction of blood and qi, blood does not nourish the tendon and vessel, joint pain and limited movement. Therefore, the treatment of this disease focuses on tonifying liver and kidney, dispelling wind and eliminating dampness, dredging the meridians and relieving pain. The main ingredients of Biqi capsule include Semen Strychni (Ma Qianzi), Pheretima (Di Long), Radix Changii (Dang Shen), Rhizoma Chuanxiong (Chuan Xiong), Radix Salviae Miltiorrhiae (Dan Shen), Radix Notoginseng (San Qi), Radix Cyathulae (Niu Xi) etc. It has the functions of nourishing qi and blood, dispelling wind and dehumidifying, promoting blood circulation, and relieving pain. Modern pharmacological research has found that,^[14]^ Biqi capsule can promote the production of tissue inhibitor of metalloproteinase-1, reduce the expression of interleukin 1 and matrix metalloproteinase-3, so as to protect articular cartilage and improve inflammation,^[15]^ it can also effectively regulate the ability of physical coordination and immune function,^[16]^ and has less adverse reactions.^[17–19]^ Therefore, we intend to evaluate the efficacy and safety of Biqi capsule in the treatment of KOA by this randomized controlled trial.

## 2 Materials and methods

### 2.1 Study design

**Figure 1:**
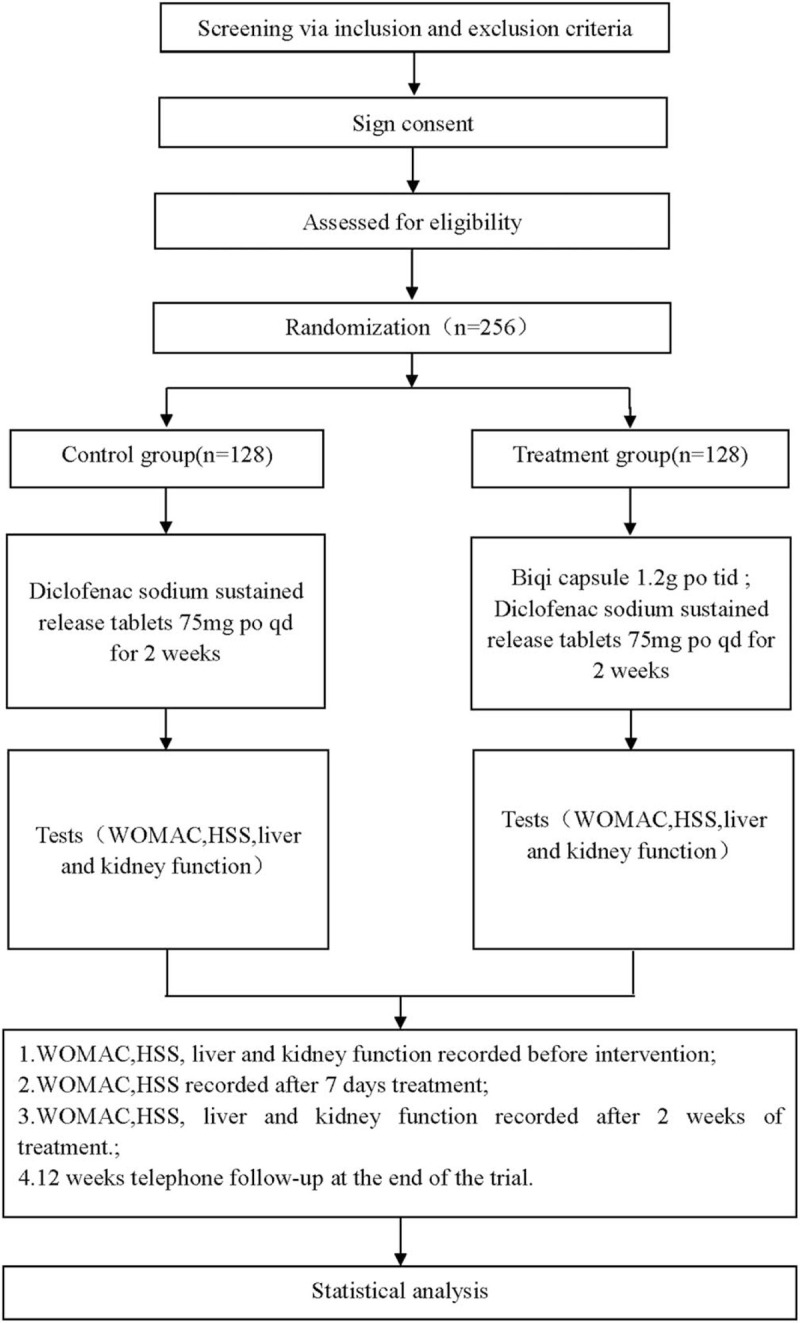
Flow diagram of the study.

This is a prospective randomized controlled trial to study the efficacy and safety of Biqi capsule in the treatment of KOA. This trial will follow the comprehensive trial reporting criteria. The flow chart is shown in Figure 1.

### 2.2 Ethics and registration

This research scheme is in line with the Helsinki Declaration and approved by the Clinical Research Ethics Committee of our hospital. This experiment has been registered he open science framework (OSF) (registration number: DOI 10.17605/OSF.IO/6HB9D). Before being randomly divided into groups, all patients are required to sign a written informed consent form and they are completely free to choose whether or not to continue the trial at any time. The flow chart of the study is shown in Figure 1.

### 2.3 Patients

Diagnostic criteria: according to the diagnostic criteria of KOA established by the Chinese Medical Association in 2010^[20]^:

1. The knee joint has frequent pain 1 month before seeing a doctor.;
2. X-ray film showed osteophyte formation;
3. Morning stiffness time ≤30minutes;
4. Accompanied by bone friction;
5. The examination of synovial fluid is consistent with KOA;
6. Age ≥40 years old. KOA can be diagnosed in a combination of 1, 2, or 1, 3, 4, 5, or 1, 3, 4, 6.

Inclusion criteria:

1. Meet the diagnostic criteria of KOA;
2. Age ≥40 years old;
3. X-ray shows Kellgren-Lawrence grade I ∼ II^[21]^;
4. All patients have signed the agreement, and the data used in the study are complete.

Exclusion criteria:

1. Combined with another organ dysfunction;
2. Suffer from other serious diseases such as malignant tumors;
3. Concomitant with serious mental illness;
4. Patients who are allergic to or have contraindications to the medication in this study;
5. Unable to understand the study protocol or unwilling participants after explanation.

Exit criteria:

1. Those who have serious adverse events and serious complications should not be further tested;
2. Those who are poor in compliance and have an impact on the judgment of effectiveness and safety;
3. Poor compliance, affecting effectiveness and safety judgment;
4. Subject requests to withdraw from the study for any reason.

### 2.4 Sample size calculation

This study was a pilot clinical trial. According to the analysis and calculation of the relevant literature published in the past 10 years, the therapeutic efficiency of western medicine is 73.81%. The effective rate of traditional Chinese medicine combined with Western medicine is 90.7%. The sample size of multiple sample rates was used to estimate the formula, if the significance test level is α=0.05, then μ_α_=1.96, 1-β=0.9, μ_β_=1.28. Sample size estimation formula for δ=π_1_-π_2_ comparison of 2 sample rates:

$$N_{1}=N_{2}=\left(\frac{\mu_{\alpha }+\mu_{\beta }}{\delta }\right)[\pi_{1}(1-\pi_{1})+\pi_{2}(1-\pi_{2})]$$

The sample size of each group was calculated to be 102 cases. Taking factors such as shedding into account, the expansion is about 20%, and 256 patients were calculated to be included. The included patients were randomly divided into control group and observation group by random number generator according to the serial number of treatments. Each group had 128 cases.

### 2.5 Study design

In this study, patients who meet the criteria will be selected through prehospital recruitment, and the patients and their families will approve the study plan and sign the informed consent form. The patients were randomly assigned to the treatment group and the control group. Among them, the treatment group will receive Biqi capsule. (Approval number: State Food and Drug Administration (SFDA) approval number Z10910026; Tianjin Darentang Jing Wanhong Pharmaceutical Co. Ltd; 0.3g × 48 tablets) Oral administration is 1.2 g (4 tablets)/time, 3 times/d Combined with diclofenac sodium sustained-release tablets (Approval number: SFDA approval number: H10980297; Beijing Novartis Pharmaceutical Co. Ltd; 75mg × 10 tablets)Oral 75 mg (1 tablet), once a day for 2 weeks; The control group was given diclofenac sodium sustained release tablets. (Approval number: SFDA approval number: H10980297; Beijing Novartis Pharmaceutical Co. Ltd; 75mg × 10 tablets) Oral 75 mg (1 tablet), once a day for 2 weeks. Patients in both groups will receive the same routine nursing, avoid tobacco, alcohol, and irritating food during medication, pay attention to rest, avoid weight-bearing on affected limbs, monitor liver and kidney function, patients with more joint effusion will be given suction treatment. If necessary, the attending physician may adjust the protocol according to the patient's condition, and all interventions will be recorded in detail for final result analysis. We will set up special drug administrators, and nurses will be responsible for the preparation of drugs and solutions. Therefore, this study is not blind to nurses, but to researchers, patients, and statisticians. The health status of each patient was evaluated before and after treatment, including efficacy and safety indicators, and all patients were followed up by telephone for 30 days. The contents of the follow-up include cardiovascular events and rehospitalization.

### 2.6 Evaluation criteria and judgment of curative effect

1. Observation index:
  a. The Western Ontario and McMaster Universities osteoarthritis index (WOMAC),^[22]^ a total of 24 items were used to evaluate the overall joint from 3 aspects: pain, stiffness, and joint function. Each item was recorded by using a visual analogue scale (VAS).^[23]^ The higher the score, the more serious the condition.;
  b. Evaluation of knee joint function by Hospital for special surgery knee score (HSS),^[24]^ the total score of HSS is 0 to 100, and the higher the score, the better the knee joint function of patients.
  c. Adverse reaction: Including abnormal liver and kidney function and any uncomfortable symptoms (such as dizziness, nausea, etc) during treatment.
  d. The clinical curative effect refers to the evaluation criteria of osteoarthritis in Chinese traditional medicine new drug clinical research guiding principle.^[25]^
  e. Significant effect: The pain symptoms disappeared or basically disappeared, joint function was basically normal, able to live and work normally, WOMAC score decreased ≥70%;
  f. Effective: The pain basically disappeared, the joint movement was slightly limited, living, and working ability was improved, and 30% ≤ WOMAC score decreased <70%;
  g. Invalid: The pain and joint function were not significantly improved compared with those before treatment, and the WOMAC score decreased by <30%.

### 2.7 Data collection and management

The data were collected according to the evaluation criteria before the beginning of treatment, 1 week after treatment and 2 weeks after the end of treatment. After 30 days of treatment, each patient was followed up by outpatient or telephone. Record in detail the reasons for the loss of follow-up for those who are unable to collect follow-up information. All data will be collected by 1 or 2 assistants in collaboration. Personal information about potential participants and registered participants will be collected, shared, and stored in a separate kept in a separate repository to protect pre -, during and post-trial confidentiality. Access to the database is limited to the researchers in this research group.

### 2.8 Statistical analysis

#### 2.8.1 Definition of dataset

Full Analysis Set: Including all the subjects who were randomly assigned to the group, took the drug at least once and visited at least once, and used the full analysis data for intent-to-treat (ITT) analysis.

Per-protocol set: Including all subjects who comply with the protocol, have no missing baseline variables, and whose primary variables are measurable.

Safety Set: They included random enrolment, at least one medication, and at least one postmedication safety interview.

#### 2.8.2 Statistical approach

In this study, SPSS25.0 statistical analysis software was used for data analysis. For measurement data in accordance with normal distribution, independent sample *t* test is used between groups, and paired sample *t* test is used within group. The results are expressed by ($\bar{X}\pm S$); For those who do not conform to normality, the rank sum test is used, and the results are expressed in the quartile; Counting data were tested by Chi-Squared test. *P* < .05 was statistically significant.

## 3 Discussion

There are many pathogenic factors of KOA, which are generally considered to be related to age, trauma, obesity, incorrect walking posture, cold, and other factors of knee joint.^[26]^ Causing cartilage degeneration and arthritis.^[27]^ With the aggravation of the disease, the activity is limited, and the longer time leads to muscle atrophy,^[28]^ which seriously affects the quality of life of patients.^[29]^ At present, Western medicine mainly uses nonsteroidal anti-inflammatory drugs or intra-articular injection of related drugs to relieve pain and improve joint function. However, due to the unstable efficacy of these drugs, the disease is caused by repeated attacks, which does not prevent the development of the disease, and the long-term use of NSAID anti-inflammatory drugs has more toxic and side effects.^[30,31]^ In recent years, traditional Chinese medicine has accumulated rich experience in the treatment of arthritis, the effect is remarkable, which can significantly improve the symptoms of patients and help to promote the recovery of joint function.^[32,33]^ The combination of traditional Chinese and Western medicine in the treatment of KOA has become the trend of clinical treatment.

Traditional Chinese medicine believes that “the knee is the house of tendons”, KOA belongs to the category of “Knee arthralgia (xibi)” and “arthralgia aggravated by cold (tongbi)” in traditional Chinese medicine. The cause of the disease is that the patient is feeble and old, deficiency of liver and kidney, loss of muscles and veins or living in wetlands for a long time, wind, cold and dampness soaking in the joints, obstruction of meridians, and collaterals leads to pain. The main disease pathogenesis is essential empty and out solid, the root cause is deficiency of liver and kidney qi and blood, the wind-cold-dampness and gas stagnation, blood stasis, phlegm coagulation is manifestation. The treatment should be replenishing qi and nourishing blood, tonifying and replenishing liver and kidney, dispelling wind and eliminating dampness and promoting blood circulation to arrest pain. Biqi capsule is widely used in the clinical treatment of rheumatoid arthritis, cervical spondylosis, scapulohumeral periarthritis, and other diseases.^[34]^ Radix Changii (Dang Shen) and Radix Salviae Miltiorrhiae (Dan Shen) are used as sovereign drug in the prescription, replenishes qi and nourishes blood, Radix Salviae Miltiorrhiae (Dan Shen) promotes blood circulation and nourishes blood; Radix Notoginseng (San Qi) promotes blood circulation and relieves pain, removes blood stasis and stops bleeding; Rhizoma Chuanxiong (Chuan Xiong) dispels wind and relieving pain, qi and blood circulation; Radix Cyathulae (Niu Xi) tonifies liver and kidney and promotes blood circulation and menstruation; Semen Strychni (Ma Qianzi) collaterals and disperses knots, reduces swelling and relieves pain; all kinds of medicines are used together to play the functions of replenishing qi and nourishing blood, dispelling wind and dehumidification, promoting blood circulation and relieving pain. At the same time, Radix Changii (Dang Shen) in Biqi capsule has anti-oxidation, anti-inflammatory, anti-cancer and many other effects^[35–37]^; Semen Strychni (Ma Qianzi) can enhance the total antioxidant capacity of organisms and improve the recovery of sensory function and motor function.^[38]^

The purpose of this randomized controlled trial is to verify that Biqi capsule can improve the clinical symptoms and joint function in patients with KOA. Since there are no standard clinical studies to evaluate the efficacy of Biqi capsule in patients with KOA, we intend to evaluate its efficacy and safety through prospective randomized controlled trials.

This study also has some limitations. Because of the short time of planned follow-up, we are unable to understand the impact of long-term results. Therefore, we may extend the follow-up time if necessary. At the same time, due to the influence of treatment, this study could not be strictly double-blind, which may affect the results to a certain extent.

## Author contributions

**Data curation:** YaZhou Zhou, WenGang Wang.

**Formal analysis:** YaZhou Zhou, WenGang Wang.

**Funding acquisition:** YaZhou Zhou.

**Resources:** WenGang Wang, Ke Tian.

**Software:** Hui Huang, Mengrui Jia.

**Study design:** YaZhou Zhou, WenGang Wang

**Supervision:** Ke Tian, Hui Huang.

**Writing – original draft:** YaZhou Zhou, WenGang Wang.

**Writing – review & editing:** YaZhou Zhou.
